# Genome-wide identification of lncRNAs associated with chlorantraniliprole resistance in diamondback moth *Plutella xylostella* (L.)

**DOI:** 10.1186/s12864-017-3748-9

**Published:** 2017-05-15

**Authors:** Bin Zhu, Manyu Xu, Haiyan Shi, Xiwu Gao, Pei Liang

**Affiliations:** Department of Entomology, China Agricultural University, 2 YuanmingyuanWest Road, Beijing, 100193 People’s Republic of China

**Keywords:** LncRNA, *Plutella xylostella*, Chlorantraniliprole, RNA-seq

## Abstract

**Background:**

Long noncoding RNAs (lncRNAs) are now considered important regulatory factors, with a variety of biological functions in many species including insects. Some lncRNAs have the ability to show rapid responses to diverse stimuli or stress factors and are involved in responses to insecticide. However, there are no reports to date on the characterization of lncRNAs associated with chlorantraniliprole resistance in *Plutella xylostella*.

**Results:**

Nine RNA libraries constructed from one susceptible (CHS) and two chlorantraniliprole-resistant *P. xylostella* strains (CHR, ZZ) were sequenced, and 1309 lncRNAs were identified, including 877 intergenic lncRNAs, 190 intronic lncRNAs, 76 anti-sense lncRNAs and 166 sense-overlapping lncRNAs. Of the identified lncRNAs, 1059 were novel. Furthermore, we found that 64 lncRNAs were differentially expressed between CHR and CHS and 83 were differentially expressed between ZZ and CHS, of which 22 were differentially expressed in both CHR and ZZ. Most of the differentially expressed lncRNAs were hypothesized to be associated with chlorantraniliprole resistance in *P. xylostella*. The targets of lncRNAs via cis- (<10 kb upstream and downstream) or trans- (Pearson’s correlation, *r* > 0.9 or < -0.9, *P* < 0.05) regulatory effects were also identified; many of the differently expressed lncRNAs were correlated with various important protein-coding genes involved in insecticide resistance, such as the ryanodine receptor, uridine diphosphate glucuronosyltransferase (UGTs), cytochrome P450, esterase and the ATP-binding cassette transporter.

**Conclusions:**

Our results represent the first global identification of lncRNAs associated with chlorantraniliprole resistance in *P. xylostella*. These results will facilitate future studies of the regulatory mechanisms of lncRNAs in chlorantraniliprole and other insecticide resistance and in other biological processes in *P. xylostella*.

**Electronic supplementary material:**

The online version of this article (doi:10.1186/s12864-017-3748-9) contains supplementary material, which is available to authorized users.

## Background

The diamondback moth (DBM), *Plutella xylostella* (L., Lepidoptera: Plutellidae), is a major insect pest of cruciferous vegetables and is considered an especially troublesome pest because of its ability to rapidly develop high resistance to insecticides used for its control [1]. To date, *P. xylostella* has developed resistance to several types of insecticides and has become one of the most resistant pests in the world [2].

Chlorantraniliprole is a new type of anthranilic diamide insecticide with a novel mode of action that activates the muscle ryanodine receptor (RyR), which controls internal calcium release in the sarcoplasmic reticulum. Activation of RyR causes rapid cessation of feeding, lethargy, muscle paralysis and, finally, insect death [3]. Because of this novel mode of action, chlorantraniliprole is very effective in controlling several orders of insects, especially lepidopteran pests. However, in recent years, *P. xylostella* has developed high levels of resistance to chlorantraniliprole in many countries, including China [4–7].

Previous studies indicate that enhanced activity of detoxification enzymes such as cytochrome P450 monooxygenase (P450), carboxylesterase (CarE) and glutathione *S*-transferases (GSTs) [8, 9] and point mutation of the target (RyR) [10–12] may be associated with chlorantraniliprole resistance in *P. xylostella*.

By using high-throughput RNA sequencing (RNA-seq) technology, Lin et al. identified 1,215 genes that may be involved in chlorantraniliprole resistance in three field-resistant *P. xylostella* strains, of which several genes were associated with calcium signaling, vascular smooth muscle contraction and cardiac muscle contraction pathways, as well as in the metabolism of xenochemicals such as insecticides [13].

Several studies have investigated mechanisms of chlorantraniliprole resistance in the past few years and many protein-coding genes have been proven to be involved in chlorantraniliprole resistance. However, research on regulatory mechanisms of these protein-coding genes remains very limited.

Most recently, microRNAs (miRNAs) have been associated with chlorantraniliprole resistance in *P. xylostella* [14]. MiRNA is a kind of endogenous small non-coding RNA (ncRNA), which regulates the expression of target genes at the transcriptional level; it has gained significant interest and popularity over the last decade [15]. Currently, another type of ncRNA, long non-coding RNA (lncRNA), has gained significant attention from researchers. Previous studies indicate that lncRNAs could show quick response to diverse stimuli or stress factors and might be involved in responses to insecticides [16, 17], so we hypothesized lncRNAs may also be associated with chlorantraniliprole resistance in *P. xylostella*.

LncRNAs are non-protein coding transcripts longer than 200 nucleotides. They were once considered inconsequential transcriptional noise. However, recent studies have shown that lncRNAs play important regulatory roles in many biological processes, including transcriptional regulation, post-transcriptional control and epigenetic processes [18, 19]. According to the position and direction of transcription in relation to protein-coding genes, lncRNAs can be further classified into several categories, such as sense, antisense, intronic and intergenic [20]. Like mRNAs, many identified lncRNAs are transcribed by RNA polymerase II, hence they are presumably capped, polyadenylated and spliced. In addition, there are also a few non-polyadenylated lncRNAs transcribed by RNA polymerase III [21]. Most lncRNAs are located only in the nucleus, but some are cytoplasmic or are in both the nucleus and cytoplasm [22].

Currently, RNA-sequencing (RNA-seq) is a very powerful approach to identify lncRNAs. In the present study, a laboratory susceptible *P. xylostella* strain and two chlorantraniliprole-resistant strains were used, and nine strand-specific RNA-seq libraries that combine rRNA removal were constructed. Four types of lncRNAs were obtained, and the relative expression of some were found to be significantly altered in chlorantraniliprole-resistant populations. These results lay a solid foundation for further study of the roles of lncRNAs in regulation of insecticide resistance in *P. xylostella*.

## Results

### Identification and characterization of lncRNAs in *P. xylostella*

**Fig. 1:**
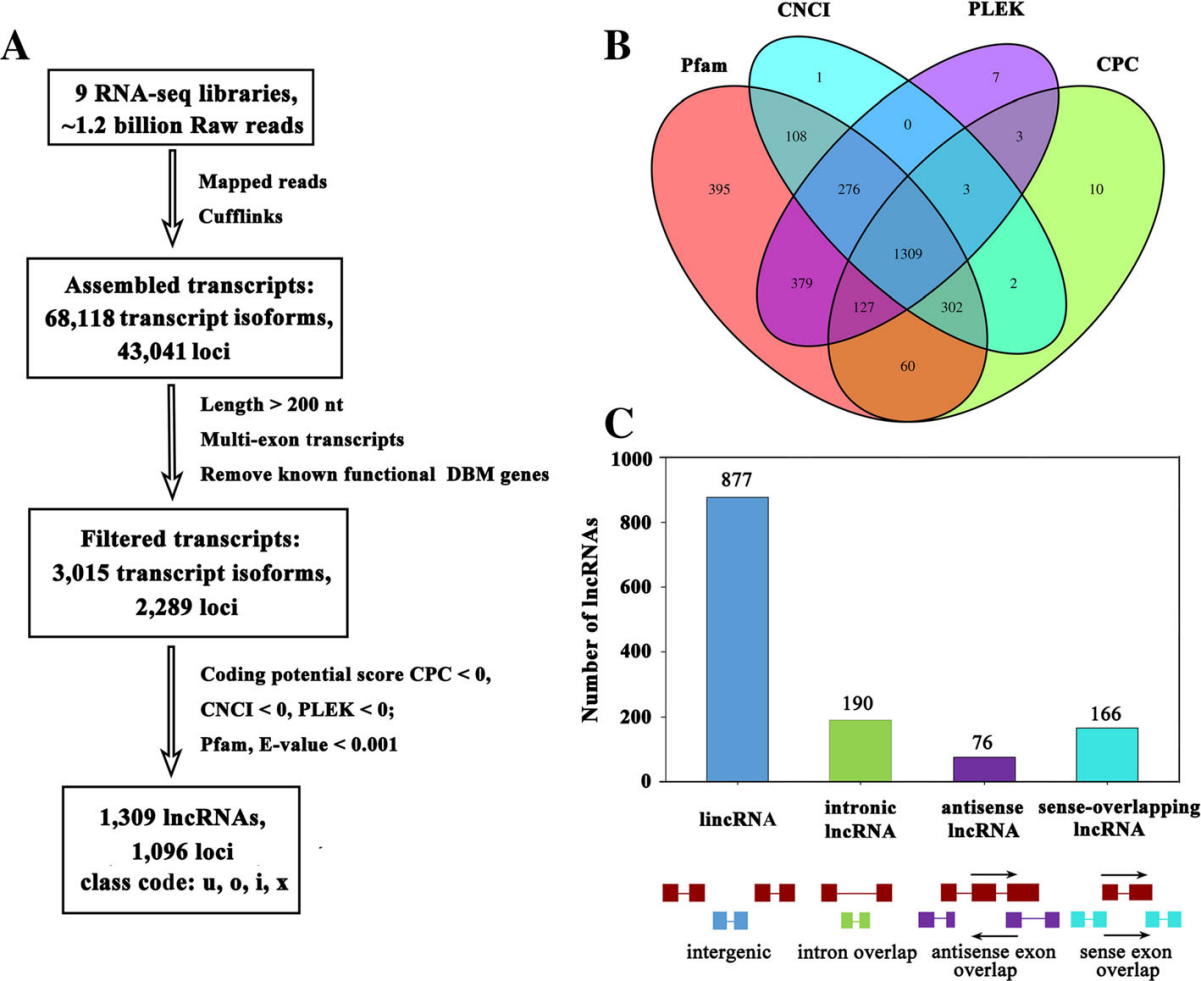
The computational pipeline for identifying lncRNAs in *P. xylostella* from RNA-seq data and their classification. **a** The lncRNA identification pipeline flowchart; **b** Coding potential analysis using the four methods; **c** The classification of identified lncRNAs, red rectangles or lines represent the exon or intron of protein-coding gene, respectively; Blue, green, purple and light blue rectangles or lines represent the exon or intron of lncRNA, respectively

High-throughput strand-specific RNA-seq was performed in the three DBM strains (CHS, CHR, ZZ), each with three biological replicates. A total of 1,198,903,526 raw reads were obtained from the nine libraries, with an average of 133 million reads per sample. After the low-quality reads were removed, 1,110,303,222 clean reads with high quality were retained (Additional file 1). Clean reads that could be mapped to the *P. xylostella* genome (GCA_000330985.1) were then used for transcript assembly and annotation. A total of 68,118 transcripts corresponding to 43,041 loci were initially generated. Then, we filtered protein-coding transcripts according to the annotated DBM reference genome (known DBM mRNAs) and transcripts with a single-exon and those that were shorter than 200 nucleotides were removed. The remaining 3,015 transcripts (corresponding to 2,289 loci) were subsequently used for protein-coding capacity prediction by using the Coding Potential Calculator (CPC) [23], Coding-Non-Coding Index (CNCI) [24], Pfam-scan (PFAM) [25] and PLEK [26] (Fig. 1b). Finally, 1,309 reliably expressed lncRNAs corresponding to 1,096 loci were obtained and were classified into four categories including ‘u’ (intergenic), ‘i’ (intronic), ‘x’ (anti-sense) and ‘o’ (sense-overlapping) according to their genomic location and referring to the neighboring genes. Specifically, the ‘u’ category contained transcripts falling in the intergenic regions between two protein-coding loci. The ‘i’ category contained transcripts falling entirely within an intron of a known protein coding gene. The ‘x’ category contained transcripts that have generic exonic overlap with a known protein coding gene on the opposite strand. The ‘o’ category contained the transcripts partial overlapping with a coding gene on the same genomic strand (Fig. 1a, b).

Most of the identified lncRNAs fell into class u, with 877 lncRNAs (67.00%), whereas 190 (14.51%), 76 (5.81%) and 166 (12.68%) lncRNAs belonged to classes i, x and o, respectively (Fig. 1c). All lncRNA sequences are listed in Additional file 2.

**Fig. 2:**
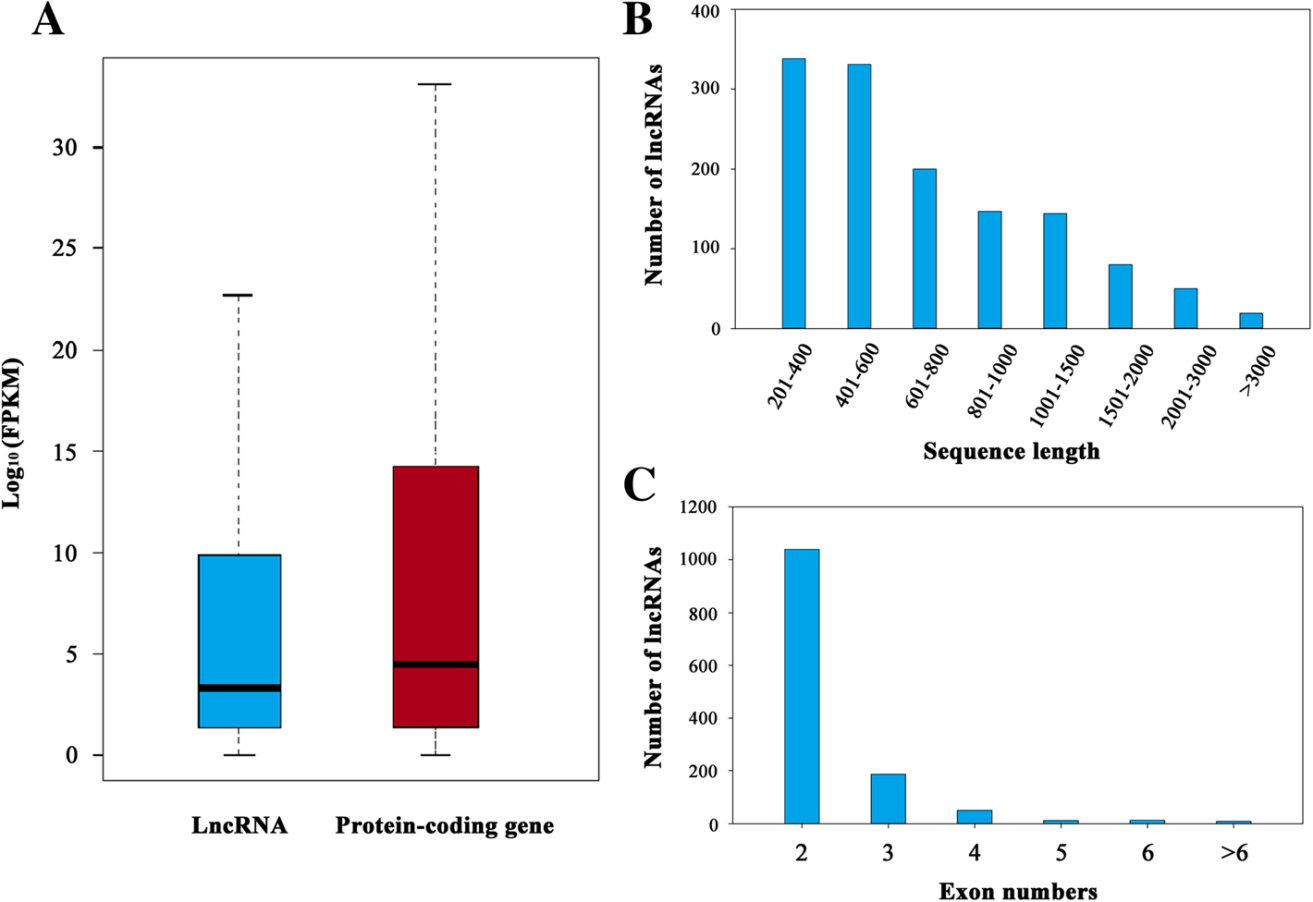
Characterization of *P. xylostella* lncRNAs. **a** Comparison of expression value (FPKM) in *P. xylostella* lncRNAs and protein coding genes; For the box-plot: midline, median; box limits, 25th percentile (first quartile) and 75th percentile (third quartile); upper whisker, min (max(x)), third quartile + 1.5× interquartile range (IQR; third-quartile minus first-quartile values); lower whisker, max(min(x)), first quartile − 1.5× IQR; **b** Size distribution of *P. xylostella* lncRNAs; **c** The distribution of exon number of lncRNAs

Previous studies in mammals have shown that the expression of lncRNAs is significantly lower than those of protein-encoding genes [27]. To determine whether *P. xylostella* lncRNAs have similar features, we measured the expression level (fragments per kilobase of exon per million fragments mapped, FPKM) of the identified lncRNAs; they generally showed a lower level of expression compared to protein-coding mRNAs (Fig. 2a).

The length and exon number of the identified lncRNAs were also analyzed. The size distribution of these lncRNAs ranged from 200 nucleotides to 7,193 nucleotides, with approximately 78% of lncRNAs shorter than 1000 nucleotides (Fig. 2b). Characterization of the genomic location revealed that the exon number of these lncRNAs ranged from 2 to 13; 1,038 (79.30%) *P. xylostella* lncRNAs had two exons, 186 (14.21%) had three exons, and 22 (1.68%) lncRNAs had more than five exons (Fig. 2c).

### Analysis of differentially expressed lncRNAs

To systematically identify chlorantraniliprole resistance-associated lncRNAs, a differential expression analysis was performed among the three strains. In total, 64 lncRNAs (45 lincRNAs, 13 sense-overlapping lncRNAs and 6 intronic lncRNAs) were identified as differentially expressed between CHR and CHS (*P* < 0.05, log_2_ (fold change) > 1), of which 34 were down-regulated and 30 were up-regulated in the CHR strain (Additional file 3, Fig. 3a, c, e). Interestingly, among these differentially expressed lncRNAs, we found 7 lncRNAs that were specifically expressed in CHS and 5 lncRNAs that were specifically expressed in CHR (Additional file 3).

**Fig. 3:**
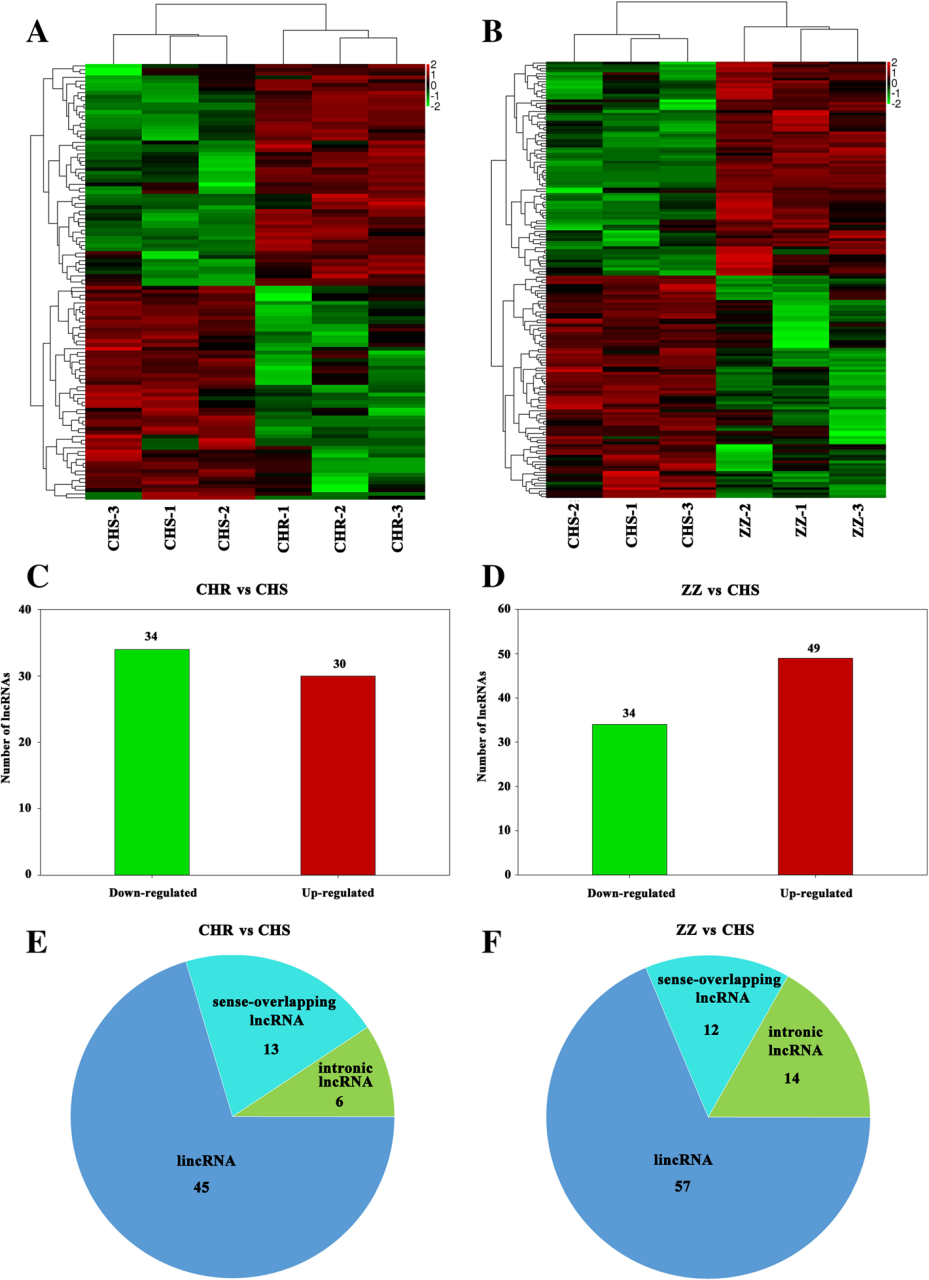
Differentially expressed lncRNAs identified among CHS, CHR and ZZ. **a** Hierarchical clustering of the differentially expressed lncRNAs between CHS and CHR; **b** Hierarchical clustering of the differentially expressed lncRNAs between CHS and ZZ; **c** Detailed statistics of differentially expressed lncRNAs between CHS and CHR; **d** Detailed statistics of differentially expressed lncRNAs between CHS and ZZ; **e** Category of differentially expressed lncRNAs between CHS and CHR; **f** Category of differentially expressed lncRNAs between CHS and ZZ

In addition, 83 lncRNAs (57 lincRNAs, 12 sense-overlapping lncRNAs and 14 intronic lncRNAs) were differentially expressed between ZZ and CHS (*P* < 0.05, log_2_ (fold change) > 1), of which 34 were down-regulated and 49 were up-regulated in ZZ (Additional file 3, Fig. 3b, d, f). Among these differentially expressed lncRNAs, 8 lncRNAs were found to be specifically expressed in ZZ and one lncRNA was specifically expressed in CHS (Additional file 3).

**Fig. 4:**
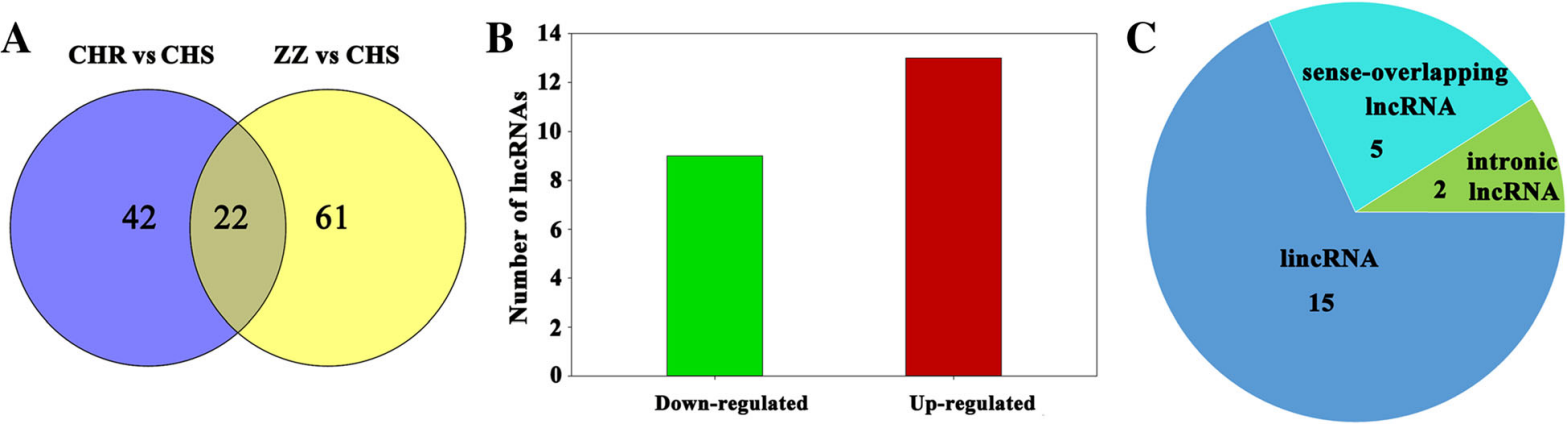
Differentially expressed lncRNAs overlapped in both CHR and ZZ. **a** Overlapped differentially expressed lncRNAs; **b** Detailed statistics of overlapped differentially expressed lncRNAs; **c** Category of overlapped differentially expressed lncRNAs

Compared to CHS, 22 lncRNAs (15 lincRNAs, 5 sense-overlapping lncRNAs and 2 intronic lncRNAs) were found to be differentially expressed in both CHR and ZZ, of which 9 were down-regulated and 13 were up-regulated in both resistant strains (Fig. 4, additional file 3). Among these lncRNAs, 4 lncRNAs were specifically expressed in both resistant strains (Additional file 3).

**Fig. 5:**
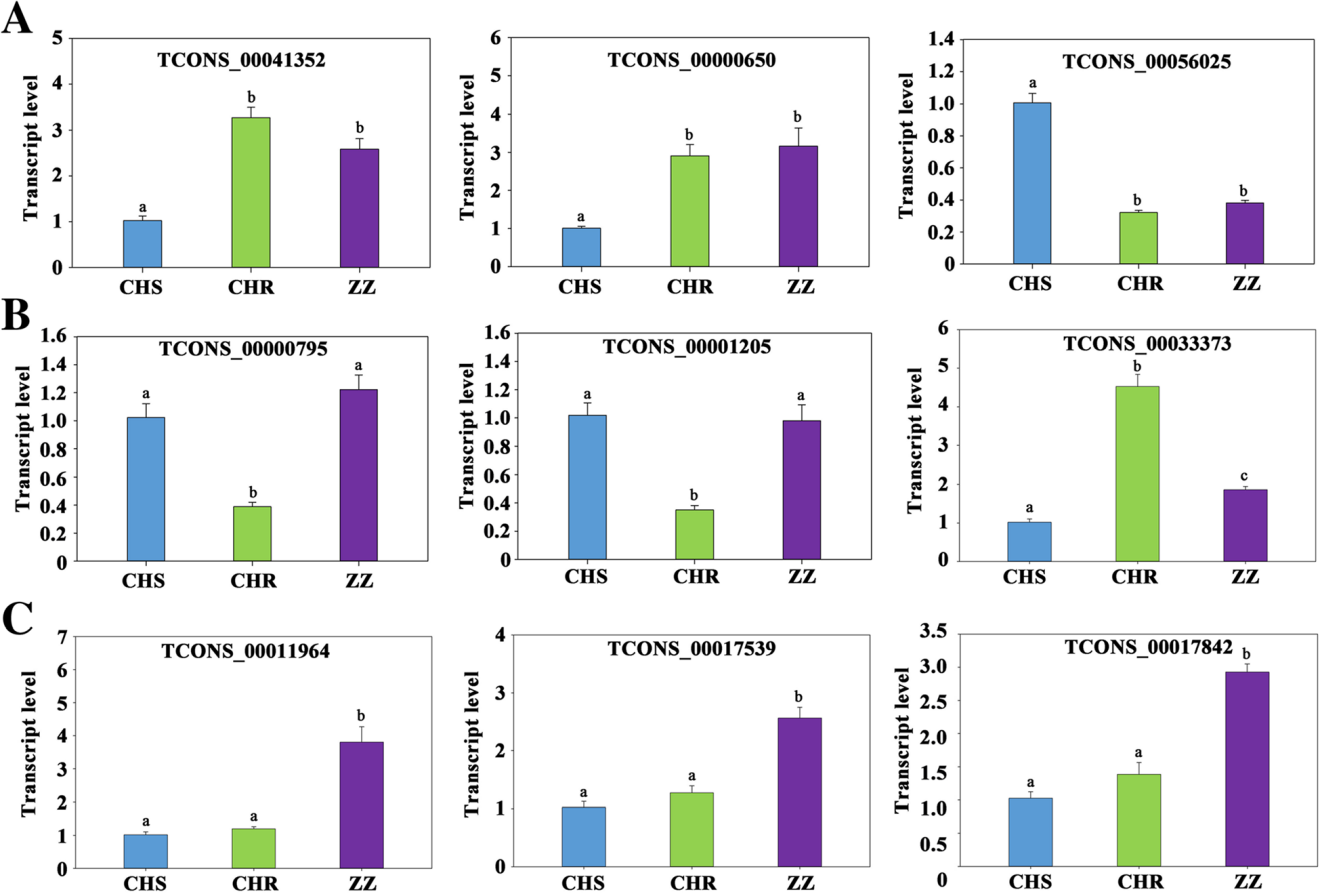
qRT-PCR validation of significantly differentially expressed lncRNAs among CHS, CHR and ZZ. Different lowercase letters represent significant differences by *t*-test (*P* < 0.05). **a**: Differentially expressed lncRNAs in both CHR and ZZ compared to those in CHS; **b**: Differentially expressed lncRNAs between CHS and CHR; **c**: Differentially expressed lncRNAs between CHS and ZZ

**Fig. 6:**
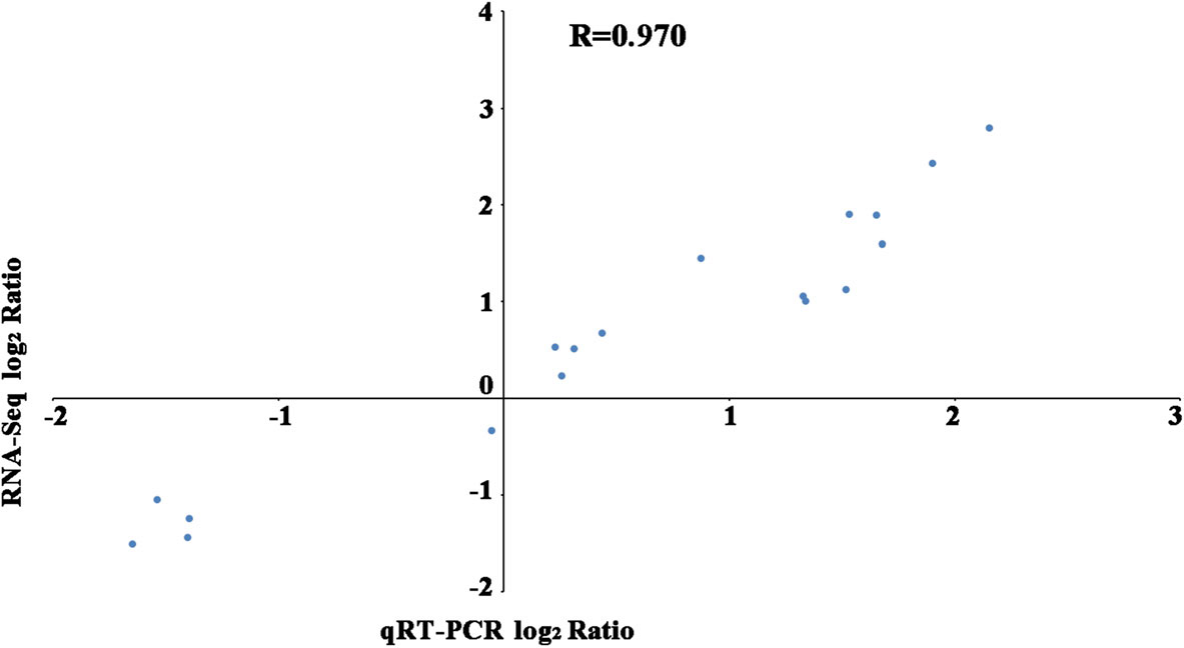
Pearson correlation between the RNA-seq and qRT-PCR data. All expression data were normalized in log_2_ ratio

To validate the RNA-seq data, three lncRNAs that were differentially expressed in both CHR and ZZ compared to CHS (TCONS_00000650, TCONS_00041352, TCONS_00056025), three lncRNAs that were differentially expressed only between CHS and CHR (TCONS_00000795, TCONS_00001205, TCONS_00033373), and three lncRNAs that were differentially expressed only between CHS and ZZ (TCONS_00011964, TCONS_00017539, TCONS_00017842) were randomly selected and their relative expression levels were quantified by qRT-PCR. The expression patterns of almost all selected lncRNAs showed a similar trend between the results of sequencing and qRT-PCR except for TCONS_00033373, which was significantly up-regulated in both the CHR and ZZ strains by qRT-PCR (Fig. 5). However, according to the sequencing results, this lncRNA was significantly up-regulated only in the CHR strain. Pearson correlation coefficient between RNA-Seq data and qRT-PCR data was 0.970, which indicates that the RNA-Seq data was highly correlated with that of the qRT-PCR (Fig. 6).

### Functional analysis of resistance-associated lncRNAs

Previous studies showed that lncRNAs may play a cis-regulatory role in mediating the expression of neighboring genes [28]. We searched for protein-coding genes 10 kb upstream or downstream of the differently expressed lncRNAs. 138 protein-coding genes (including 19 overlapped protein-coding genes) were found close to 64 differentially expressed lncRNAs between CHR and CHS, and 177 protein-coding genes (including 26 overlapped protein-coding genes) were found close to 83 differentially expressed lncRNAs between ZZ and CHS (Additional file 4). Among these neighboring protein-coding genes, only the ATP-binding cassette sub-family G member 1 (ABCG1) gene overlapped with TCONS_00022357 (down-regulated in ZZ) was previously associated with insecticide resistance [29] (Additional file 4). When Gene Ontology (GO) analysis of the neighboring genes was performed, we found that most of the differently expressed lncRNAs between CHS and CHR or between CHS and ZZ may play a role in binding-associated functions in cis mode, because most of the neighboring genes were annotated as binding-related GO terms, such as metal ion binding, ATP binding, DNA binding. Meanwhile, neighboring genes were also enriched in transcription in the Biological Process category (BP) and the nucleus, integral component of membrane, cytoplasm in Cellular Component (CC) categories (Additional file 4).

Many studies have also shown that lncRNAs can function as trans-regulatory elements. Thus, potential targets of the differently expressed lncRNAs in trans-regulatory relationships were predicted using a co-expression analysis. Pearson’s correlation test (*r* > 0.9 or < -0.9, *P*-value < 0.05) was used in this study. For differently expressed lncRNAs and protein-coding genes between CHS and CHR, a total of 802 interaction relationships (580 positive and 222 negative correlations) were detected (Additional file 5). Similarly, for differently expressed lncRNAs and protein-coding genes between CHS and ZZ, a total of 2420 interaction relationships (1,823 positive and 597 negative correlations) were obtained (Additional file 5), which was approximately 3-fold of that between CHS and CHR. This huge difference may be caused by the obvious differences between CHR and ZZ. The former was a laboratory resistant strain selected from CHS with only chlorantraniliprole, while the later came from the field with quite different genetic background and was exposed to many different insecticides over a long time period.

Interestingly, 2 lncRNAs, TCONS_00013329 and TCONS_00056155, were found to be co-expressed with the ryanodine receptor (the target of chlorantraniliprole; TCONS_00013329 was significantly up-regulated in both resistant strains and TCONS_00056155 was significantly down-regulated only between CHS and CHR. Many lncRNAs are also co-expressed with various protein coding genes involved in insecticide resistance, such as UDP-glucuronosyltransferase (UGTs), cytochrome P450, esterase, glutathione *S*-transferase (GSTs), ATP-binding cassette transporter (ABC), heat shock protein (HSP) and cuticle protein (Additional file 5). GO enrichment analysis based on the correlated target protein-coding genes was also performed. All correlated protein-coding genes of the differently expressed lncRNAs in the two comparison groups were enriched in similar GO terms, such as metal ion binding, ATP binding, DNA binding, zinc ion binding, serine-type endopeptidase activity and RNA binding in Molecular Function (MF); transcription, regulation of transcription, DNA integration, innate immune response and oxidation-reduction process in Biological Process (BP); and nucleus, cytoplasm, integral component of membrane in Cellular Component (CC) (Additional file 5).

## Discussion

In recent years, the vast majority of studies on lncRNA have been conducted in mammals, especially in humans. Studies of insect lncRNAs are still in preliminary stages. With the rapid development of high-throughput techniques, a batch of lncRNAs has been identified in several insect species, such as *Drosophila melanogaster* [30, 31], *Apis mellifera* [32], *Apis cerana* [33], *Anopheles gambiae* [34], *Aedes aegypti* [35], *Nilaparvata lugens* [36], *Bombyx mori* [37] and *P. xylostella* [16], of which only *N. lugens* and *P. xylostella* are agricultural pests.

The publication of the *P. xylostella* genome in 2013 [38] benefits the identification of lncRNAs considerably. In a recent annotation of the DBM genome in NCBI, 707 transcripts were annotated as ncRNAs. In addition, Etebari et al. [16] also identified 3844 DBM long intergenic non-coding RNAs (lincRNAs) from RNA deep-sequencing data downloaded from the NCBI Sequences Read Archive.

In the current study, a total of 1,309 lncRNAs belonging to four types were identified from 9 strand-specific RNA-seq libraries, of which 77 could be blast-searched in the NCBI database. These were annotated as DBM ncRNAs and shared a similar locus, including 67 lincRNAs, 5 intronic lncRNAs, 1 sense-overlapping lncRNA and 4 antisense lncRNAs (Additional file 2). In addition, 190 lncRNAs partially overlapped (shared similar loci in the same scaffold of the DBM genome) with the lincRNAs identified by Etebari et al. [16] according to the transcript location supplied in their research, but because of differences in the library building method and the RNA-seq data used in our research, some of the overlapped lncRNAs were re-classified to other lncRNA types rather than lincRNA. Specifically, 4 of these overlapped lncRNAs were re-classified as antisense lncRNAs, including TCONS_00003280 (overlapped with lincRNA_210, a lincRNA named by Etebari et al. [16]), TCONS_00006143 (overlapped with lincRNA_379), TCONS_00008192 (overlapped with lincRNA_495) and TCONS_00030950 (overlapped with lincRNA_1763); 18 were re-classified as intronic lncRNAs, such as TCONS_00018632 (overlapped with lincRNA_1075) and TCONS_00019803 (overlapped with lincRNA_1140); and 8 were re-classified as sense-overlapping lncRNAs, such as TCONS_00010817 (overlapped with lincRNA_658) and TCONS_00011269 (overlapped with lincRNA_673) (Additional file 2). Moreover, there were 17 overlapping lncRNAs between the 190 lincRNAs identified by Etebari et al. [16] and the 77 lncRNAs annotated in the NCBI (Additional file 2). Therefore, 1,059 novel lncRNAs were identified in this research. The number of putative lncRNAs detected in this study was less than that reported by Etebari et al. [16], mainly because all the transcripts that contained only one exon were retained in their research, but only multiple exon transcripts were used for lncRNA identification in the present study.

In addition, to our knowledge, this is the first application of rRNA removal and strand-specific RNA sequencing to study the DBM transcriptome. This method allows non-polyA transcripts to be obtained, which is an obvious advantage compared to polyA enrichment sequencing [39]. Strand information was also included in our sequencing data, which made it easy to distinguish sense transcripts from antisense transcripts. As a result, anti-sense lncRNAs were identified in *P. xylostella* for the first time. Different types of lncRNAs may play their roles in different way, so a detailed classification of lncRNAs would help us to further understand their various functions [20].

Differently expressed lncRNAs among CHS, CHR and ZZ were analyzed in the present study, most associated with chlorantraniliprole or other insecticide resistance. The CHR strain was established from CHS by successive selection with chlorantraniliprole and has been reared under the same laboratory conditions as CHS; all 64 differentially expressed lncRNAs between them are likely to be associated with chlorantraniliprole resistance. The ZZ strain was collected from the field and had developed middle to high levels of resistance to several commonly used insecticides besides chlorantraniliprole, such as beta-cypermethrin, abamectin, spinosad and indoxacarb (unpublished data from a local plant protection station). Each of these insecticides has a distinctive mode of action. Therefore, the 83 differentially expressed lncRNAs in the ZZ strain likely result from the comprehensive effects of these different insecticides as well as from other environmental factors.

When the differentially expressed lncRNAs in CHR and ZZ strains were put together, 22 of them overlapped. These common differentially expressed lncRNAs are very likely involved in chlorantraniliprole resistance because they were differentially expressed in both laboratory-selected and field-collected resistant strains, and both of these strains were resistant to chlorantraniliprole. However, due to the complexity of insecticide resistance mechanisms in the ZZ strain, these 22 lncRNAs may also reveal resistance mechanisms common to other insecticides besides chlorantraniliprole. Interestingly, four of these 22 lncRNAs were specifically expressed in the two resistant strains. We speculated that their transcription might be induced by long-term exposure to chlorantraniliprole, and these four lncRNAs may play key roles in chlorantraniliprole resistance in *P. xylostella*. The other 42 lncRNAs differentially expressed in the CHR strain may also be involved in chlorantraniliprole resistance. Though this hypothesis was not supported by data from the field resistant strain, these lncRNAs should not be ignored in the further study of the mechanisms of chlorantraniliprole resistance in *P. xylostella*. We suspected that most of these unique differentially expressed lncRNAs may play specific roles in resistance only to chlorantraniliprole. Meanwhile, the lncRNAs that were differentially expressed only in the ZZ strain are more likely to be involved in resistance to other insecticides besides chlorantraniliprole.

Notably, several of their overlapping lincRNAs for the differently expressed lncRNAs identified in the present study have been found to be involved in insecticide resistance in *P. xylostella* by Etebari et al. [16]. For example, lincRNA_2514 overlapped with TCONS_00044413 is involved in chlorpyrifos, fipronil and Bt resistance; lincRNA_1623 overlapped with TCONS_00028420 in Bt and fipronil resistance; lincRNA_494 overlapped with TCONS_00008143 in Bt resistance; and lincRNA_1624 overlapped with TCONS_00028420 in chlorpyrifos resistance, respectively [16]. Our finding of the involvement of these lncRNAs in chlorantraniliprole resistance increases the possibility that these lncRNAs play some important roles in insecticide resistance regulation.

To further study the roles of lncRNAs possibly associated with chlorantraniliprole resistance, we predicted the potential function of the differently expressed lncRNAs using cis and trans methods. In the cis prediction, numerous protein-coding genes were found within 10 kb upstream or downstream from the concerned lncRNAs, most of which may play a role in binding-associated activity. In the trans prediction, potential targets of the differently expressed lncRNAs were predicted using co-expression analysis and many target protein-coding genes involved in insecticide resistance were identified, indicating that lncRNAs may regulate insecticide resistance by directly affecting these target genes. For example, XM_011567276, annotated as cytochrome P450 6B6, was co-expressed with 11 lncRNAs (TCONS_00044883, TCONS_00026933, TCONS_00019595, TCONS_00032346, TCONS_00065690, TCONS_00019598, TCONS_00008336, TCONS_00037191, TCONS_00007659, TCONS_00065766 and TCONS_00052631). Previous studies showed that one lncRNA could regulate multiple protein-coding genes and vice versa [40]. Here, these 11 lncRNAs may collectively regulate the expression of cytochrome P450 6B6, thus enhancing the metabolism of insecticides in *P. xylostella*. In fact, Peng et al. [41] have identified a set of lncRNAs highly correlated with the expression of P450 in mouse liver during maturation. Interestingly, the ryanodine receptor, the main target of chlorantraniliprole, was also found to be co-expressed with two lncRNAs (TCONS_00013329 and TCONS_00056155), and these lncRNAs may directly control the expression of the ryanodine receptor to mediate chlorantraniliprole resistance. In addition to this, several binding terms were identified as enriched GO terms for the target mRNAs in both comparison groups. LncRNAs play important roles in regulating biological functions through various mechanisms that are not fully understood; these proposed mechanisms include regulation based on RNA-protein interactions as well as RNA-RNA interactions and RNA-DNA interactions [42]. Here, binding terms were identified as enriched GO terms for the correlated mRNAs in both comparison groups, and it is very likely that lncRNAs may act primarily through these interactions.

## Conclusions

In the current study, 1,309 lncRNAs were identified from 9 RNA-seq libraries of *Plutella xylostella*, including 877 intergenic lncRNAs, 190 intronic lncRNAs, 76 anti-sense lncRNAs and 166 sense-overlapping lncRNAs. In addition, several lncRNAs showed significant expression changes in the two chlorantraniliprole-resistant strains; some were identified as co-expressed with several genes involved in insecticide resistance, especially the ryanodine receptor, the target of chlorantraniliprole. These results provide solid bases for further investigation of the roles of lncRNAs in regulation of chlorantraniliprole and other insecticide resistance and in other biological processes in *P. xylostella*.

## Methods

### Insects

The susceptible DBM strain (CHS) was collected in the vegetable fields of Beijing and maintained in our laboratory without any insecticide treatments for more than 10 years. The chlorantraniliprole-resistant strain (CHR) was derived from the CHS strain by uninterrupted selection with chlorantraniliprole for more than 70 generations. The Zhangzhou strain (ZZ) was collected in the vegetable fields of Zhangzhou, Fujian province, southeastern China in 2015; before sequencing, the ZZ strain was selected with chlorantraniliprole for two generations in our laboratory. Moreover, the toxicity of chlorantraniliprole to the CHS, CHR and ZZ populations was tested using a leaf dipping method as described elsewhere [43]; the CHR and the ZZ strains showed 65-fold and 42-fold resistance to chlorantraniliprole, respectively, compared to the susceptible CHS strain [44]. All stages of *P. xylostella* were maintained at 27 ± 1 °C, with an RH of 40–60% for radish seedlings (*Raphanus sativus* L.) and a photoperiod of 16:8 h (L:D). *P. xylostella* adults were provided with 10% (W/V) honey solution and were allowed to lay eggs on radish seedlings.

### RNA extraction, library preparation and sequencing

Thirty 3rd instar *P. xylostella* larvae were collected in a PE tube as one sample. Trizol Reagent (Invitrogen, Carlsbad, CA, USA) was used to isolate total RNA according to the manufacturer’s instructions. RNA degradation and contamination were assessed on 1% agarose gels and RNA concentration was measured using a NanoDrop 2000 (Thermo Fisher Scientific Inc, USA).

Library construction and RNA-Seq were performed by the OE Biotechnology Corporation (Shanghai, China). Total RNA from 9 samples (three independent biological replicates for each of the CHS, CHR and ZZ strains) with RNA integrity number (RIN) values above 8 were used to construct RNA-Seq libraries using the TruSeq stranded total RNA preparation kit with Ribo-Zero Gold (Illumina, San Diego, CA, USA) according to the manufacturer’s instructions. Sequencing was performed on the Illumina HiSeq™2500 and 150 bp paired-end reads were generated.

### Bioinformatics analysis

Raw data in FASTQ format were first processed using the NGS QC Toolkit [45]. In this step, clean data (clean reads) were obtained by removing reads containing adapters, reads containing poly-N, low quality reads (lower than 20) and contaminants from the raw data. At the same time, the Q20, Q30 and GC content of the clean data were calculated. All the downstream analyses were based on clean data with high quality.

The latest reference genome and gene model annotation files of *P. xylostella* (GCA_000330985.1) were downloaded from the NCBI FTP site (ftp://ftp.ncbi.nlm.nih.gov/genomes/Plutella_xylostella/). The clean reads from each library were first aligned to the DBM genome using TopHat [46], then the mapped reads were assembled using Cufflinks in a reference-based approach [47].

### Identification of lncRNAs

The assembled transcripts were annotated using the Cuffcompare program from the Cufflinks package [46]. According to the annotations of the DBM genome sequence, the known protein-coding transcripts as well as the rRNA, tRNA, snRNA, snoRNA, pre-miRNA and pseudogenes were first removed. Meanwhile, transcripts with single exons and those that were shorter than 200 bps were also excluded from further non-coding analysis. The coding potential for the remaining transcripts was calculated by using CPC [23], CNCI [24], Pfam [25] and PLEK [26]. Transcripts revealing coding potential with a CPC score > 0, CNCI score > 0, PLEK_score > 0 and Pfam-scan > 0.001 were all removed. The identified lncRNAs were finally separated into four types using the class code module in Cuffcompare [46].

### Differential expression analysis

The number of reads mapped to each lncRNA and protein-coding transcript was determined using HT-Seq software (http://www-huber.embl.de/users/anders/HTSeq/doc/index.html) [48]. The expression level of each transcript was measured by FPKM. Differential expression analysis was performed using the DESeq R package [49]. The DESeq package implements the negative binomial model to compute differentially expressed transcripts. *P*-value < 0.05 and |log_2_ (fold change) | > 1 were considered as significantly differential expression. Hierarchical Clustering was performed using the Agilent GeneSpring GX software (version 11.5.1).

### Quantitative real-time PCR (qRT-PCR)

Quantitative real-time PCR was performed to experimentally validate the relative expression levels of the identified lncRNAs. Total RNA from the same samples used for deep sequencing were used for the first-strand cDNA synthesis using PrimeScript™ RT reagent Kit with gDNA Eraser (Perfect Real Time) (Takara Biotechnology, Dalian, China) per the manufacturer’s instructions. qRT-PCR analysis was carried out using SYBR Premix Ex Taq (Takara Biotechnology, Dalian, China). Each reaction was performed on an ABI 7500 Real Time PCR system (Applied Biosystems) with three biological replicates. The relative expression levels of lncRNAs and protein coding genes were calculated using the 2^–ΔΔCt^ method [50]. Ribosomal protein L32 mRNA was used as a reference gene. Pearson correlation coefficient between qRT-PCR data and RNA-Seq data was calculated to validate RNA-Seq experiments. All primers used in this study are listed in Additional file 6.

### Functional analysis of resistance-associated lncRNAs

None of the DBM lncRNAs are functionally annotated in current databases. Thus, we estimated their functions based on the functional annotations of their related protein-coding genes. In cis, we searched for all the protein-coding genes 10 kb upstream or downstream of the differently expressed lncRNAs. In trans, we used the expression levels of the differently expressed lncRNAs and protein-coding genes to analyze their co-expression relationships. Pearson correlation with *P*-value < 0.05 and Pearson’s correlation coefficients (*r* > 0.9 or < -0.9) were considered as correlated expression. All identified neighboring genes and co-expressed genes were used for the GO enrichment analysis, respectively. A *P* < 0.05 was considered statistically significant. The GO analysis was divided into Molecular Function, Biological Process and Cellular Component. The results allowed us to predict the functional classification in which the target genes of the differentially expressed lncRNAs were enriched.

## Additional files

Additional file 1: Table S1. Summary of RNA-seq data. (DOCX 13 kb)

Additional file 2: Table S2. LncRNAs identified in this study. (XLSX 395 kb)

Additional file 3: Table S3. Differentially expressed lncRNAs between CHS and CHR as well as CHS and ZZ. (XLSX 26 kb)

Additional file 4: Table S4. Protein-coding genes 10 kb upstream or downstream of the differently expressed lncRNAs. (XLSX 45 kb)

Additional file 5: Table S5. Protein-coding genes co-expressed with the differently expressed lncRNAs. (XLSX 256 kb)

Additional file 6: Table S6. All primers used in this study. (XLSX 9 kb)

## Abbreviations

ABC: ATP-binding cassette transporter
CarE: Carboxylesterase
FPKM: Fragments per kilobase of exon per million fragments mapped
GSTs: Glutathione S-transferases
HSP: Heat shock protein
LncRNA: Long noncoding RNA
P450: Cytochrome P450 monooxygenase
qRT-PCR: Quantitative real-time reverse transcription polymerase chain reaction
RNA-seq: RNA sequencing
RyR: Ryanodine receptor
UGTs: Uridine diphosphate glucuronosyltransferase
