# Supplementary material for: Genome-wide identification of lncRNAs associated with chlorantraniliprole resistance in diamondback moth Plutella xylostella (L.)
Source: BMC Genomics. 2017 May 15;18:380. doi: 10.1186/s12864-017-3748-9 (PMC5433093; doi:10.1186/s12864-017-3748-9)
Supplement: Supplementary file 1 — Summary of RNA-seq data. (DOCX 13 kb) [file 12864_2017_3748_MOESM1_ESM.docx]

**Summary of RNA-seq data**

| **Sample** | **Raw reads** | **Raw bases** | **Clean reads** | **Clean bases** | **Valid ratio**  **(base)** | **Q30**  **(%)** | **GC content**  **(%)** |
| --- | --- | --- | --- | --- | --- | --- | --- |
| Sample_R1 | 121368932 | 15171116500 | 111231074 | 13897230400 | 91.60% | 93.78% | 48.00% |
| Sample_R2 | 131418650 | 16427331250 | 120893014 | 15104574472 | 91.94% | 93.99% | 48.00% |
| Sample_R3 | 159987548 | 19998443500 | 147290124 | 18402762183 | 92.02% | 94.09% | 48.00% |
| Sample_S1 | 121691792 | 15211474000 | 112111798 | 14007512800 | 92.08% | 94.12% | 48.00% |
| Sample_S2 | 132380172 | 16547521500 | 121545952 | 15186042200 | 91.77% | 93.91% | 49.00% |
| Sample_S3 | 120664776 | 15083097000 | 112135946 | 14011341499 | 92.89% | 94.35% | 48.50% |
| Sample_Z1 | 129348174 | 16168521750 | 122334562 | 15286950632 | 94.54% | 95.13% | 48.00% |
| Sample_Z2 | 133730490 | 16716311250 | 125967528 | 15740624676 | 94.16% | 94.86% | 47.00% |
| Sample_Z3 | 148312992 | 18539124000 | 136793224 | 17091275479 | 92.19% | 94.15% | 47.50% |
